# Complete genome sequence of a vampire bat-related rabies virus obtained by metagenomics from a patient with encephalitis of unknown etiology, French Guiana

**DOI:** 10.1128/mra.00514-24

**Published:** 2024-10-04

**Authors:** Véronique Hourdel, Charlotte Balière, Jessica Vanhomwegen, Angela Brisebarre, Quentin Grassin, Jean-Claude Manuguerra, Hatem Kallel, Magalie Demar, Laurent Dacheux, Valérie Caro

**Affiliations:** 1 Institut Pasteur, Université Paris Cité, Laboratory for Urgent Response to Biological Threats (CIBU), Environment and Infectious Risks (ERI) Unit, Paris, France; 2 Service de Réanimation Polyvalente, Centre Hospitalier de Cayenne, Cayenne, French Guiana; 3 Laboratoire de Biologie Médicale, Centre Hospitalier de Cayenne, Cayenne, French Guiana; Katholieke Universiteit, Leuven, Belgium

**Keywords:** rabies, French Guiana, complete genome, metagenomics, encephalitis, diagnosis, Illumina, next generation sequencing, bat, human

## Abstract

We report the complete genome sequence of a rabies virus obtained by direct metagenomics from the cerebellum of a gold panner who died of unknown encephalitis in French Guiana. Phylogenetic analysis exhibited a close genetic relationship with vampire bat-related isolates, confirming the second case of human rabies identified in this territory.

## ANNOUNCEMENT

Rabies remains a major but still neglected tropical disease, with an estimated number of 59,000 fatal human cases worldwide each year (1). Rabies virus (RABV) is the principal etiological agent, belonging to the species *Rabies lyssavirus* within the genus *Lyssavirus*, family *Rhabdoviridae* (order *Mononegavirales*) (2). Apart from domestic dogs, other mammals such as bats can act as natural hosts and vectors. In French Guiana, an overseas territory localized in the northern part of South America, rabies transmitted by hematophagous bats (mainly the *Desmodus rotundus* species) remains endemic. Regular cases are detected in cattle, domestic carnivores, or even bats (3, 4). In 2008, the first confirmed human case of vampire rabies was detected (5). We describe here the RABV genome sequence of the second human Guyanese case, whose diagnosis was based on direct metagenomics.

A post-mortem cerebellum biopsy (lab ref 2024-6-10) was collected from a deceased patient presenting with encephalitis of unknown etiology in late February 2024 in Cayenne Hospital, French Guiana. This patient first developed symptoms while working on an illegal gold mining site in the Amazon rainforest. Nucleic acids were extracted with a DNA/RNA Microprep plus kit (Zymo Research). Total RNA was used for reverse transcription using random hexamer primers and the SuperScript IV First-Strand cDNA Synthesis kit (Thermofisher Scientific) and amplified with the Quantitect Whole Transcriptome kit (Qiagen). All steps were performed according to the manufacturer’s instructions. A sequencing-ready library was prepared using the TruSeq DNA PCR-Free prep kit (Illumina) and sequenced on a NextSeq2000 system (Illumina) generating nearly 14.4 million of 300-bp single-end length reads (88.2% of unique reads and 12.8% of duplicate reads).

Nearly 77 million contigs were generated and identified against the RVDB database (v25.0) using Microseek, retaining only contigs with a length of 100 nucleotides (6). Among them, a contig (9,010 nt) related to *Lyssavirus rabies* species was identified. A second dedicated workflow built on Galaxy@Pasteur (a Galaxy platform hosted by Institut Pasteur) was subsequently performed, combining cleaning, *de novo* assembly, and mapping (CLC Assembly Cell, Qiagen, v5.1.10) (7–10). A final consensus was generated with the nearly 8 million reads retained and manually edited using SnapGene (v7.2) before visualization using Tablet (11).

This complete RABV genome was 11,922 nucleotides in length, with 294.80× coverage on average and 18,078 (0.22%) mapped reads. The five canonical genes were present, encoding the nucleoprotein (N, 1,353 nt, 450 aa), phosphoprotein (P, 894 nt, 297 aa), matrix protein (M, 609 nt, 202 aa), glycoprotein (G, 1,575 nt, 524 aa), and RNA polymerase (L, 6,387 nt, 2,128 aa). The sequences of leader and trailer extremities were complete, with 58 and 69 nucleotides in length, respectively (checking done after alignment with genetically close and available complete genomes). The transcription initiation signal AACA and the transcription termination polyadenylation (TTP) sequence TGA7 were observed for all the genes, except for the G gene, which presented the AGA7 motif for TTP.

**Fig 1:**
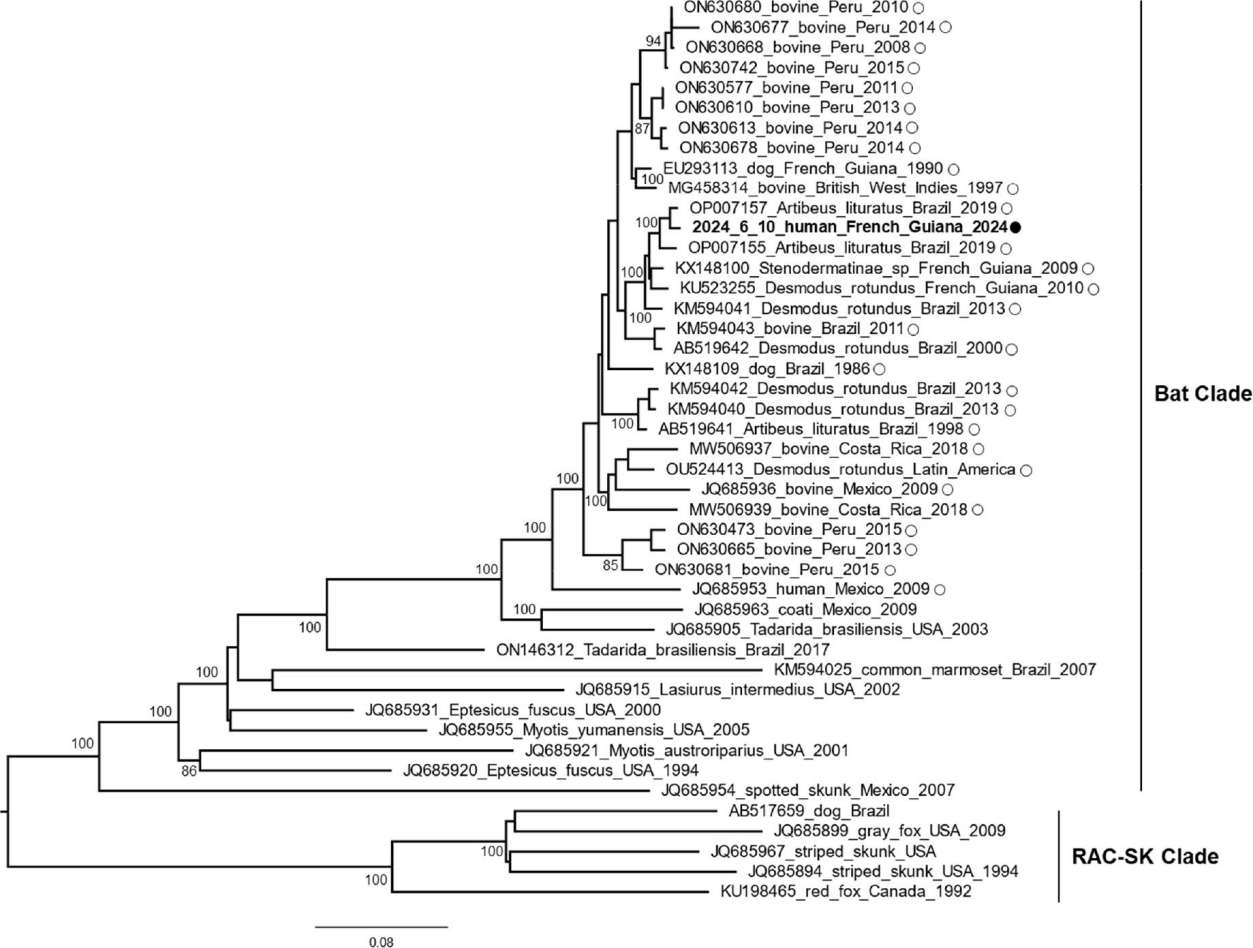
Phylogenetic analysis of the RABV strain (2024-6-10) obtained from the cerebellum of a deceased patient presenting with encephalitis of unknown etiology in French Guiana and different representative American strains. The tree was based on the multiple sequence alignment corresponding to the concatenation of the five ORFs (10,815–10,818 nt), performed with ClustalW (v2.1) (14) implemented in Galaxy@Pasteur (9). The tree was constructed using the ML approach based on the generalized time-reversible model proportion of invariable sites plus gamma-distributed rate heterogeneity (GTR+I+Γ4) utilizing Subtree Prune-and-Regraft (SPR) branch-swapping, as estimated in PhyML 3.0 (12) with Smart Model Selection, also implemented in Galaxy@Pasteur. The robustness of individual nodes was estimated using 100 bootstrap replicates. All tools were run with default parameters unless otherwise specified. The ML phylogenetic tree was visualized with FigTree (http://tree.bio.ed.ac.uk/). Only bootstrap values ≥80 are indicated for the major branches. The scale bar indicates nucleotide substitutions per site. Each sequence denomination contains the GenBank accession number, the nature of the infected species, and the country and year of isolation, where applicable. Rabies virus isolates associated with vampire bats are indicated by an open circle. The isolate (2024_6_10_RABV_GUY) described in this study is indicated in bold, with a black circle. RAC-SK, raccoon–skunk (rabies virus associated with the raccoon and skunk host animals).

Maximum likelihood (ML) phylogenetic analysis was performed on the five concatenated open reading frames (ORFs) with different representative American strains using PhyML (12), and genetic analysis confirmed that the virus clustered together with vampire bat-related isolates within the Bat Clade (Fig. 1) (13).

## Data Availability

The complete genome sequence of the vampire bat-related rabies virus from French Guiana was deposited in GenBank under accession number PP764242 and BioProject accession number PRJNA1108161.
